# Garlic Powder Supplementation Improves Growth, Nonspecific Immunity, Antioxidant Capacity, and Intestinal Flora of Chinese Mitten Crabs (*Eriocheir sinensis*)

**DOI:** 10.1155/2022/6531865

**Published:** 2022-11-07

**Authors:** Ruoyu Zhou, Jinsong Liu, Xueyan Shi, Chunsheng Fu, Ying Jiang, Ruiqiang Zhang, Yanping Wu, Caimei Yang

**Affiliations:** ^1^College of Animal Science and Technology, College of Veterinary Medicine, Zhejiang Agricultural and Forestry University, Hangzhou, Zhejiang 311300, China; ^2^Key Agricultural Research Institute of Green Animal Health Products of Zhejiang Province, Zhejiang Vegamax Biotechnology Co., Ltd., Anji, Zhejiang 313300, China; ^3^Institute of Animal Nutrition, Huai'an Kangda Feed Co., Ltd., Xuyi, Jiangsu 211700, China

## Abstract

This study was conducted to survey the effects of garlic powder on growth performance, nonspecific immunity, antioxidant capacity, and intestinal flora structure of Chinese mitten crabs. Altogether, 216 crabs which originally weigh 20.71 ± 0.13 g were randomly allocated into three treatment groups with 6 replicates of 12 crabs per replicate. The control group (CN) was fed a basal diet, while the other two groups were fed the basal diet supplemented with 1000 mg/kg (GP1000) and 2000 mg/kg (GP2000) garlic powder, respectively. This trial lasted 8 weeks. The results showed that the supplementation of garlic powder improved the final body weight, weight gain rate, and specific growth rate of the crabs (*P* < 0.05). Meanwhile, in serum, better nonspecific immune was confirmed by the enhancement of phenoloxidase and lysozyme levels, with the improvement of phosphatase activities in GP1000 and GP2000 (*P* < 0.05). On the other hand, the levels of total antioxidant capacity, glutathione peroxidases, and total superoxide dismutase in serum and hepatopancreas were increased (*P* < 0.05) while malondialdehyde content declined (*P* < 0.05) as the garlic powder was added to the basal diet. And, catalase in serum also shows an increase (*P* < 0.05). In both GP1000 and GP2000, genes related to antioxidant and immunity, for instance, *Toll-like receptor 1*, *glutathione peroxidase*, *catalase*, *myeloid differentiation factor 88*, *TuBe*, *Dif*, *relish*, *crustins*, *antilipopolysaccharide factor*, *lysozyme*, and *prophenoloxidase* mRNA expression levels, were increased (*P* < 0.05). The abundance of *Rhizobium* and *Rhodobacter* was reduced by adding garlic powder (*P* < 0.05). This study indicated that dietary addition of garlic powder promoted growth, enhanced nonspecific immunity and antioxidant capacity, activated Toll pathway, IMD pathway, and proPO system, increased antimicrobial peptide expression, while simultaneously improving the intestinal flora of Chinese mitten crabs.

## 1. Introduction

Chinese mitten crab (*Eriocheir sinensis*) is both an indigenous and economically significant aquatic economic species. As one of the economic aquatic products, it relies on its rich and delicious traits to attract the majority of consumers and win a good reputation. For this reason, it is not only very popular with consumers but also can reach an annual output of 756, 877 metric tons according to the China Fishery Statistical Yearbook in 2019 [1]. With the expansion of the Chinese mitten crab industry, the consumers also pay more attention to its quality and safety issues. Excessive demand leads to high breeding density, and a series of diseases are caused by water pollution caused by external pollutants, for instance, pesticides and organic pollutants [2]. In order to improve the immunity and disease resistance of crustaceans and improve their growth performance and health status, rational use of immune enhancers is of great significance to the development of the aquaculture industry. It is essential to find an effective method to improve the antioxidant capacity and immune capacity of the Chinese mitten crabs. For the sake of the sustainable and sound economic growth in aquaculture, in the meantime, to fit in eco-friendly growth models, we shift to seek solutions in nonantibiotic strategy.

Garlic is the underground bulb of *Allium* in *Liliaceae*. It possesses a strong and spicy smell of garlic, and its components are rich in protein, oligosaccharides, and polysaccharides, as well as fat and minerals. As a potential substitute for antibiotics as a feed additive, garlic powder contains several compounds such as allicin, S-allyl cysteine, and allyl disulfide, which have antioxidant, antiviral, and antimicrobial effects, improve immune ability and antioxidant capacity, and activate the expression of immune-related genes [3]. The use of garlic in fisheries has been shown to have excellent advantages in killing bacteria, fungi, and pathogenic bacteria [4]. Garlic has become increasingly popular as a growth promoter in aquaculture because of its ability to boost immune system activity and ward off disease [5, 6]. Garlic is used in a variety of different aquaculture species, such as rainbow trout (*Oncorhynchus mykiss*), spotted grouper (*Epinephelus coioides*), catfish (*Clarias gariepinus*) [7]. In addition to its various superior functions, garlic has become popular because of its low cost, easy processing into feed, and little environmental impact.

Chinese mitten crabs are equipped to live in a complex environment full of bacteria, fungi, or viruses mainly owing to the strong innate immune response, which is different from mammals [8]. Transcriptome analysis indicated that the Chinese mitten crab regulated innate immunity to pathogens through Toll, immune deficiency (IMD), Janus kinase (JAK) signal transduction and transcription activator (STAT), and mitogen-activated protein kinase (MAPK) pathways [9]. Hepatopancreas in crustaceans is not only an important part of the digestive system but also an important role in the degradation of toxic substances.

Currently, limited knowledge is reported to evaluate the regulatory role and mechanism of garlic powder on Chinese mitten crabs. Therefore, this study was carried out for the purpose of revealing the effect of dietary garlic powder supplementation on growth performance, nonspecific immunity, antioxidant capacity, and intestinal flora of Chinese mitten crabs.

## 2. Materials and Methods

### 2.1. Moral Statement

All the crabs involved in this experiment were handled in accordance with the licensing standards for the care and use of laboratory animals established by the Animal Experimentation Ethics Committee of Zhejiang A&F University.

### 2.2. Experimental Design, Diet, and Management

The experimental crabs were provided by Jiangsu Jinkangda Group Aquatic Experiment Base. The crabs were raised in an aquarium (each 678 L), and each aquarium was a replicate. 7 days prior to the beginning of the experiment, planted *Alternanthera philoxeroides*, after waiting for *Alternanthera philoxeroides* to survive, water quality match the conditions for the crabs to live, and the water temperature is 24.0~29.0°C during the experiment, dissolved oxygen >7.0 mg·L^−1^, total nitrogen <0.01 mg·L^−1^. After a week of temporary rearing, altogether, 216 crabs were randomly selected with similar sizes and average body weight (20.71 ± 0.13 g) for the experiment and were randomly allocated into three treatment groups with 6 replicates of 12 crabs per replicate. And make sure that there were six male crabs and six female crabs in each tank. The control group (CN) was fed a basal diet, while the other two groups were fed the basal diet supplemented with 1000 mg/kg (GP1000) and 2000 mg/kg (GP2000) garlic powder, respectively [10, 11]. The garlic powder was obtained from Zhejiang Vegamax Biotechnology Co., Ltd. (China). The content of total garlic polysaccharide in garlic powder is ≥40.0%, and the content of effective garlic polysaccharide is ≥25.0%. The experiment lasted for eight weeks. The experimental diet was provided by the relevant feed manufacturer and it is extruded feed. The formula and general composition of the basic diet are shown in Table 1. It should be noted that 1000 mg/kg and 2000 mg/kg garlic powder premix ought to make to lay the foundation for the subsequent proportioning feed. The daily feeding was cast twice for feeding amount for about 5% of the total weight of the crab, one in the morning at 7:30 (1/3) and the other at 4:30 in the evening (2/3). The daily ration was adjusted according to the feed surplus of the previous day. The fully aerated aquaculture water was added, changed 1/3 to 1/2 of the water every day, and kept inflating during the breeding period.

### 2.3. Sample Collection

At the end of the experiment, the crabs in each aquarium were counted and weighed. Two male crabs from each aquarium were randomly selected. The experimental crabs were anesthetized on ice. Hemolymph was collected with 1 mL syringe after being washed with anticoagulant. Hemolymph samples from 2 random crabs in the aquarium were stored in the same centrifuge tube, and then, the hemolymph was centrifuged at 5000 × *g* and 4°C for 15 min, and the upper serum was collected and stored at -80°C for testing. The hepatopancreas and the intestinal contents were collected in sterile centrifugal tubes on an aseptic operating table and stored in a refrigerator at -80°C for testing after quick-freezing with liquid nitrogen.

### 2.4. Growth Performance

In the final week of the feeding experiment, the entire crabs were deprived of food for 24 hours before weighing and collecting. Parameters are calculated as follows:

(1) $$\begin{array}{c} \text{Survival rate }\left(\text{SR},\%\right)=100\times \frac{\text{survival number}}{\text{initial number of crabs}},\\ \text{Weight gain rate }\left(\text{WGR},\%\right)=100\times \frac{\text{final body weight}-\text{initial body weight}}{\text{initial body weight}},\\ \text{Specific growth rate }\left(\text{SGR},\%\;{\text{day}}^{-1}\right)=100\times \frac{\ln\left(\text{final body weight}\right)-\ln\left(\text{initial body weight}\right)}{\text{days}},\\ \text{Feed conversion ratio }\left(\text{FCR},\text{g}/\text{g}\right)=\frac{\text{feed consumed}}{\text{final weight}-\text{initial weight}}. \end{array}$$

### 2.5. Serum Immunity

The phenoloxidase (PO) activity was spectrophotometrically measured by using a 96-well flat-bottled plate using L-DOPA as substrate, and the enzyme activity was measured at 490 nm with the help of a microplate reader. Enzyme activity was defined as an increase of 0.001 per minute in OD490 by one enzyme activity unit under test conditions, so PO's unit is defined as U/min.

Employ the lysozyme (LZM) assay kit (Nanjing Jiancheng Institute of Biological Engineering, China) while following the usage instructions to detect LZM activity. The detection principle of LZM activity is that by hydrolyzing peptidoglycan on the cell wall of bacteria, the bacteria are lysed and the concentration is reduced, resulting in enhanced light transmittance. Therefore, the content of LZM can be estimated according to the change of light transmittance. The substrate used in the method mentioned above is lyophilized micrococcus Lysodeikticus at a concentration of 0.25 mg/mL. 0.2 mL of collected serum or standard LZM solution (2.5 *μ*g/mL) was mixed with 2 mL of lysococcus working solution. A spectrophotometer (UV-1200, Shanghai MADA Instrument Co., Ltd.) was used to read the absorbance reduction value of the reaction solution at 530 nm, and the reaction continued. Finally, LZM activity was calculated according to the absorbance of the standard LZM solution, in which the unit is *μ*g/mL.

Alkaline phosphatase (AKP) and acid phosphatase (ACP) activities were determined using ACP and AKP detection kits (Nanjing Jiancheng Institute of Biological Engineering). Both AKP and ACP can decompose benzene disodium phosphate to generate free phenols. Under alkaline conditions, phenols react with 4-amino-antipyrazoline and are oxidized by potassium ferricyanide to generate red quinone derivatives. Enzyme activity can be measured according to the depth of red. The activities of AKP and ACP were defined as follows: at 37°C, 1 U of 1 mg phenol was produced by the reaction of every 100 mL serum with the substrate for 15 min. The units of AKP and ACP are both U/100 mL.

### 2.6. Antioxidant Capacity

The hepatopancreas tissues were added into precooled saltwater at a mass/volume ratio of 1 : 9, then broken by ultrasonic wave, and centrifuged at 4000 × *g* at 4°C for 10 min, and the supernatant was carefully collected for determination.

The total antioxidant capacity (T-AOC) indicates total antioxidant compounds, which can reduce Fe^3+^ to Fe^2+^, and Fe^2+^ can form a stable complex with film materials, so the level of antioxidant capacity can be measured by colorimetry. Prepare the reagent according to the instructions and mix thoroughly with the serum sample to be tested in proportion. Take a water bath at 37°C for 30 minutes and leave the mixture for 10 minutes. The absorbance value of each tube was measured under the condition of 520 nm wavelength and 1 cm light diameter with the zero-adjusted spectrophotometer.

The determination of glutathione peroxidase (GPx) also used spectrophotometer colorimetry for enzymatic reaction and color reaction. At 37°C, the concentration of GSH in the reaction system was reduced by 1 *μ*mol/L to one GPx enzyme activity unit per 0.1 mL serum for 5 min.

The activity of total superoxide dismutase (T-SOD) was determined with a corresponding detection kit (Nanjing Jiancheng Institute of Biological Engineering, China). The basic principle is that the xanthine-xanthine oxidase system produces superoxide radical O^2-^, and both SOD and WST-1 catalyze decomposition into hydrogen peroxide (H_2_O_2_) and oxygen (O_2_).

Considering that the addition of ammonium molybdate can quickly stop the decomposition process of hydrogen peroxide by catalase (CAT), the activity of CAT can be calculated by measuring the change of the light yellow complex produced by the interaction between residual hydrogen peroxide and ammonium molybdate at 405 nm. The units of T-AOC, GPx, T-SOD, and CAT are all U/mL.

To determine the content of malondialdehyde (MDA), thiobarbituric acid and MDA were bathed in water at 95°C for 40 min, cooled, and centrifuged for 10 min (3500 r/min). The absorbance value of the supernatant was determined at 532 nm. MDA in the lipid degradation product of peroxide can condense with thiobarbituric acid to form a red product with a maximum absorbance of 532 nm. Using this characteristic, the content of MDA can be determined. The unit of MDA is nmol/mL. All the methods mentioned above were successful in collecting scientific data according to the manufacturer's protocols of the test kit (purchased from Nanjing Angle Gene Bioengineering Co., Ltd.).

### 2.7. Hepatopancreas Gene Expression

With the help of TRIzol total RNA extraction reagent (No. 9108, Takara, Japan), total RNA was extracted from hepatopancreas. The amount and concentration of total RNA were determined spectrophotometrically with the aid of Nano-300 (No. 1053, ALLSHENG, China). The RNA solution was stored in DEPC water, which is ultrapure water treated with diethyl pyrocarbonate and autoclaved, free of impurities of RNA, DNA, and protein. The next step is to reverse transcribe the RNA to cDNA and store it at -20°C in preparation for subsequent real-time quantitative PCR (RT-qPCR). RT-qPCR amplification reactions were performed using the SYBR kit (RR420A, Takara, Japan). Primer sequences of target genes are shown in Table 2.

### 2.8. Intestinal Flora Structure

DNA from intestinal contents was extracted using a kit (Qiagen GmbH, Hilden, Germany), and genomic DNA integrity was assessed by 1% agarose gel electrophoresis. DNA was diluted to 1 ng/*μ*L with sterile water and stored at -80°C for subsequent sequencing. The 16S rRNA gene containing the V3-V4 region was amplified by PCR using primers designed according to conserved regions in the sequence, forward primer (5′-ACTCCTACGGGAGGCAGCA-3′) and reverse primer (5′-GGACTACHVGGGTWTCTAAT-3′). Sequencing and related biological analyses were performed on the Illumina HiSeq platform of Novogene (China). Operational taxonomic unit (OTU) generally indicates merging sequences from one or more samples based on an artificial sequence similarity threshold, and sequences with a similarity higher than the threshold will be merged into one OTU. In studies of 16S rRNA gene diversity, 97% sequence similarity is often used as the OTU threshold. In the alpha diversity analysis, we refined OTU into multiple indicators, including ACE (abundance-based coverage estimator), Chao1, observed species, Shannon, and Simpson. ACE and Chao1 indexes are calculated values of sample flora richness, and the larger the value is, the higher the sample flora richness will be. Observed species refers to the number of directly observed species. Shannon represents the total number of categories and their proportion in the sample. The higher the community diversity is, the more uniform the species distribution is, and the larger the Shannon index is. Simpson describes the diversity and evenness of species distribution in a community. The lower the Simpson index is, the higher the bacterial diversity is. The diversity of beta was analyzed by principal coordinate analysis (PCoA) to investigate the structural changes of microbial communities between samples. The group abundance was statistically compared at phylum and genus levels. PCoA can present the visual coordinates of the similarity or difference of the research data and is used to study the similarity or difference of the sample community composition.

### 2.9. Data Analysis

Data were assessed by analysis of variance (ANOVA) with the quadratic in SPSS version 22.0. GraphPad Prism 8.0 was used for the analysis of the data. A multiple comparison Duncan test was used to examine significant differences among all groups and a *P* value < 0.05 was considered significant.

## 3. Results

### 3.1. Growth Performance

As shown in Table 3, compared with the CN group, final body weight, WGR, and SGR in GP1000 and GP2000 were significantly increased (*P* < 0.05), and FCR showed no significant difference (*P* > 0.05). No significant differences (*P* > 0.05) were seen in SR, final body weight, WGR, and SGR in GP1000 compared to GP2000.

### 3.2. Serum Immunity

**Figure 1:**
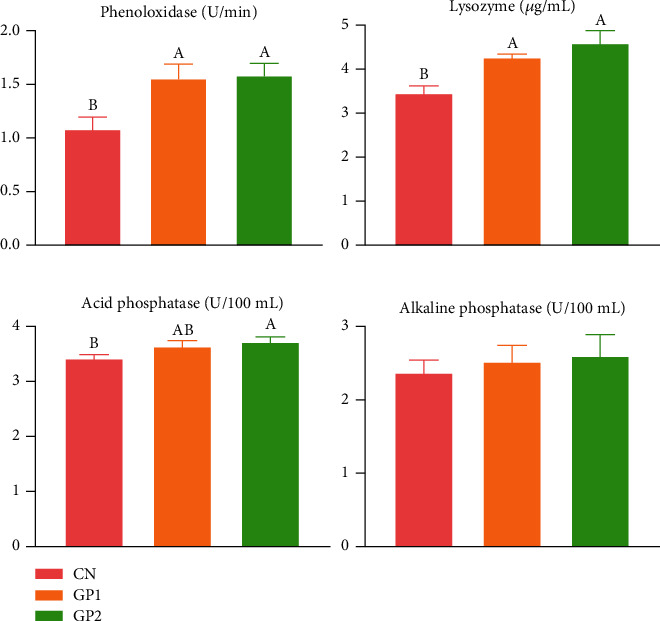
Effects of garlic powder inclusion on the serum immune indexes of Chinese mitten crabs. Bars assigned with different superscripts are significantly different (*P* < 0.05); CN represents the control group that Chinese mitten crabs fed a basic diet; GP2 represents the group that the Chinese mitten crabs fed with 1000 mg/kg garlic powder in addition to the basic diet. GP2 represents the group that the Chinese mitten crabs fed with 2000 mg/kg garlic powder in addition to the basic diet.

Dietary 1000 and 2000 mg/kg garlic powder supplementation significantly (*P* < 0.05) improved serum PO and LZM activities in Chinese mitten crabs (Figure 1). The ACP activity in GP2000 was significantly (*P* < 0.05) higher than that in CN. Although AKP activities in GP1000 and GP2000 increased, they did not show a significant difference (*P* > 0.05).

### 3.3. Antioxidant Status in Serum and Hepatopancreas

**Figure 2:**
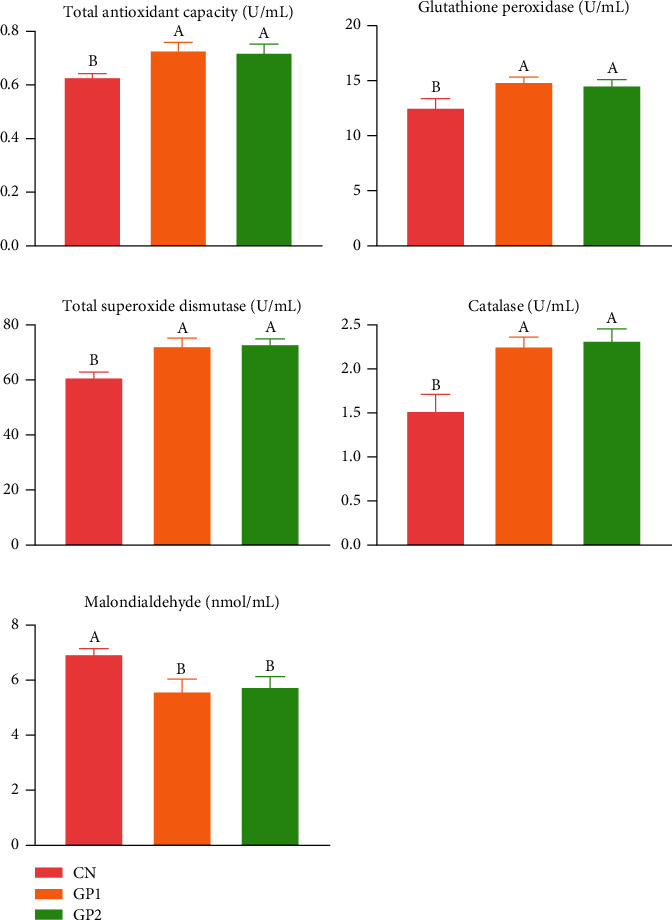
Effects of garlic powder inclusion on the antioxidant status of serum in Chinese mitten crabs. Bars assigned with different superscripts are significantly different (*P* < 0.05); CN represents the control group that Chinese mitten crabs fed a basic diet; GP2 represents the group that the Chinese mitten crabs fed with 1000 mg/kg garlic powder in addition to the basic diet; GP2 represents the group that the Chinese mitten crabs fed with 2000 mg/kg garlic powder in addition to the basic diet.

**Figure 3:**
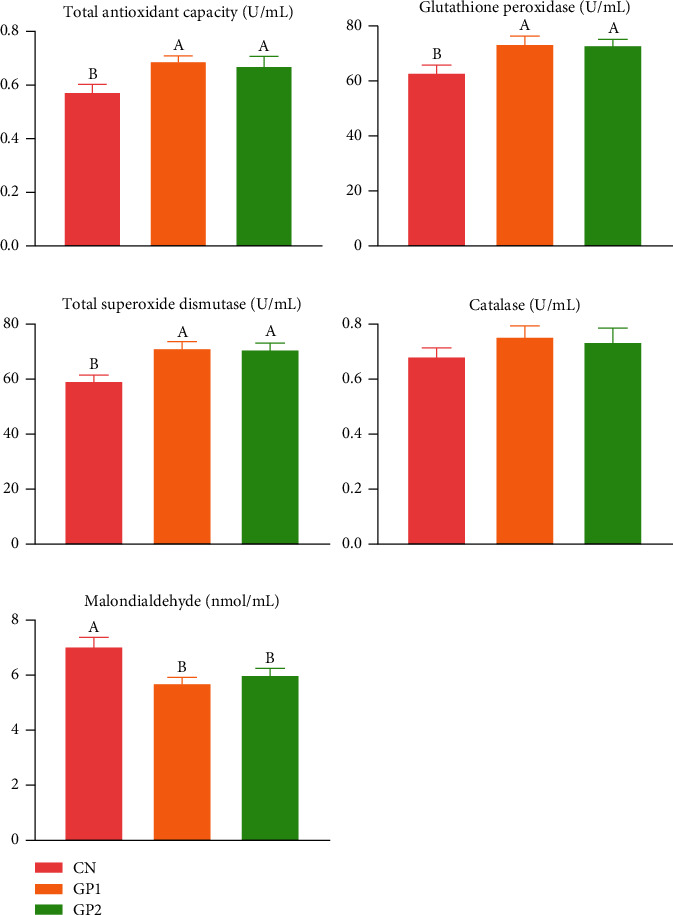
Effects of garlic powder inclusion on the antioxidant status of hepatopancreas in Chinese mitten crabs. Bars assigned with different superscripts are significantly different (*P* < 0.05); CN represents the control group that Chinese mitten crabs fed a basic diet; GP2 represents the group that the Chinese mitten crabs fed with 1000 mg/kg garlic powder in addition to the basic diet; GP2 represents the group that the Chinese mitten crabs fed with 2000 mg/kg garlic powder in addition to the basic diet.

As indicated in Figure 2, dietary supplementation with 1000 and 2000 mg/kg garlic powder exhibited significant (*P* < 0.05) enhancement in T-AOC, GPx, T-SOD, and CAT in serum. GP1000 and GP2000 both showed a significant decrease in MDA contents (*P* < 0.05). In the determination of antioxidant indexes of hepatopancreas, as shown in Figure 3, the changing trend of T-AOC, GPx, T-SOD, and MDA levels was consistent with that in serum (*P* < 0.05), and the difference was that CAT has no significant change (*P* > 0.05) after adding garlic powder.

### 3.4. Antioxidant and Immune-Related Gene Expression

**Figure 4:**
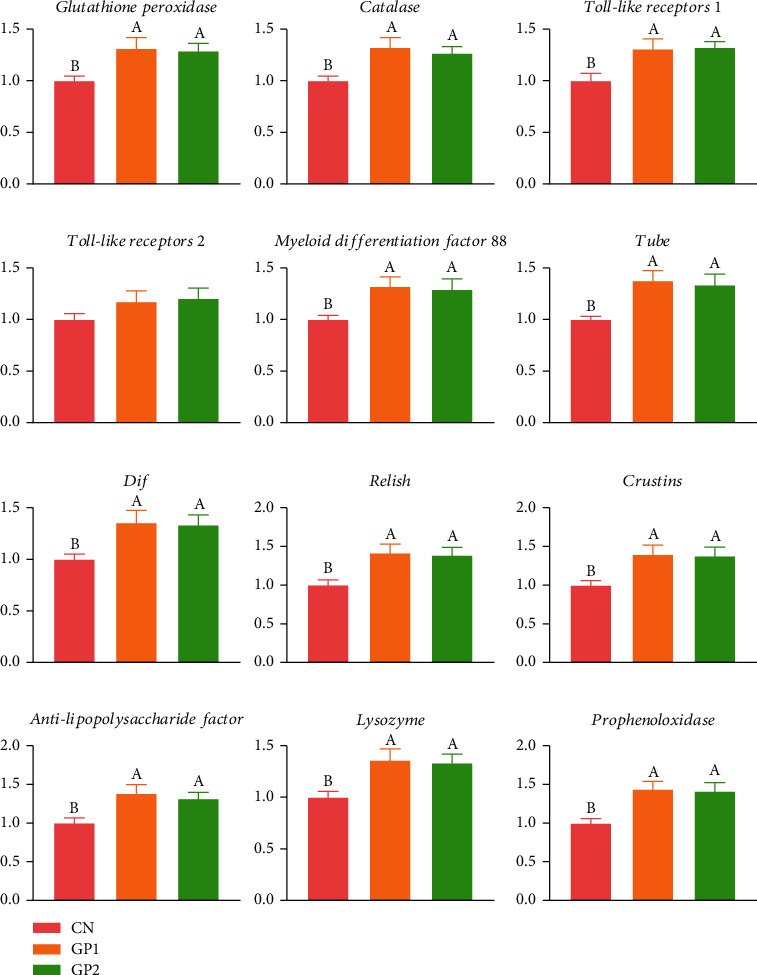
Effects of garlic powder inclusion on antioxidant and immune-related gene expression of hepatopancreas in Chinese mitten crabs. Bars assigned with different superscripts are significantly different (*P* < 0.05); CN represents the control group that Chinese mitten crabs fed a basic diet; GP2 represents the group that the Chinese mitten crabs fed with 1000 mg/kg garlic powder in addition to the basic diet; GP2 represents the group that the Chinese mitten crabs fed with 2000 mg/kg garlic powder in addition to the basic diet.

As illustrated in Figure 4, the relative expression levels of *GPx*, *CAT*, *Toll-like receptor 1*, *MyD88*, *TuBe*, *Dif*, *relish*, *crustins*, *ALF*, *LZM*, and *proPO* of crabs fed with 1000 and 2000 mg/kg garlic powder diet were significantly (*P* < 0.05) higher than that in the CN group.

### 3.5. Intestinal Flora Structure

**Figure 5:**
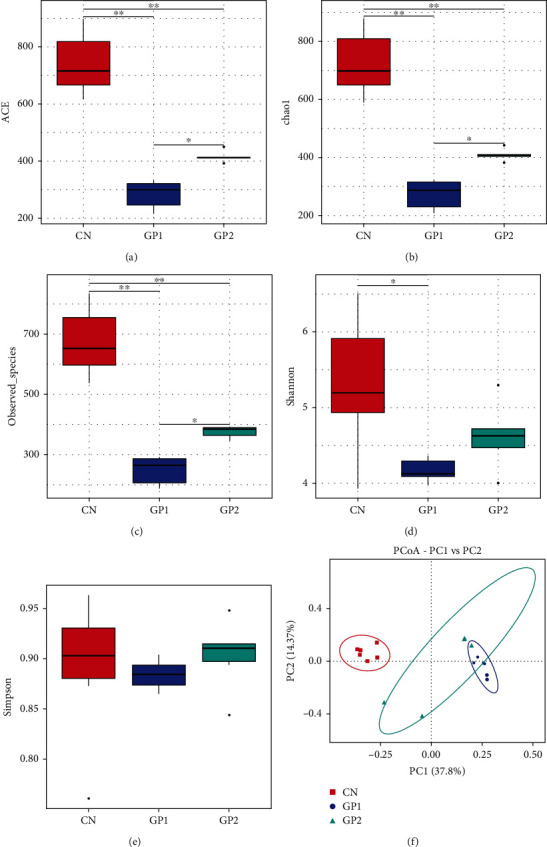
Effects of garlic powder inclusion on microflora of intestinal content in Chinese mitten crabs. Bars assigned with different superscripts are significantly different (*P* < 0.05). (a) ACE; (b) chao1; (c) observed species; (d) Shannon; (e) Simpson; (f) PCoA.

Firstly, for alpha diversity analysis, as shown in Figure 5, ACE, Chao1, and observed species indexes in GP1000 and GP2000 were lower than that in CN. The results indicated that garlic powder significantly decreased the intestinal microbial richness (*P* < 0.05). Shannon and Simpson indexes were significantly decreased in GP1000 and GP2000 compared with the CN (*P* < 0.05), which showed that garlic powder mainly decreased the intestinal microbial community species diversity of Chinese mitten crabs.

Secondly, beta diversity analysis was used to demonstrate the differences between samples by using the method of principal coordinate analysis (PCoA). The composition of the intestinal flora of Chinese mitten crabs in GP1000 and GP2000 supplemented with garlic powder was similar and significantly changed compared with the control group (*P* < 0.05). It is indicated that the species composition of GP1000 and GP2000 was different from CN.

**Figure 6:**
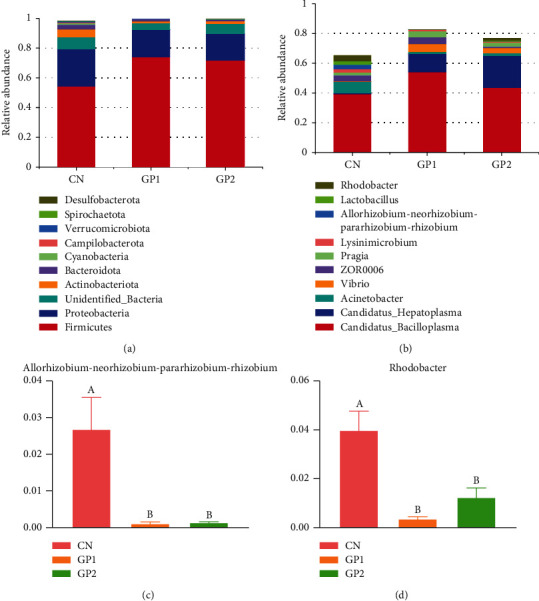
Effects of garlic powder inclusion on microflora of intestinal content in Chinese mitten crabs. Bars assigned with different superscripts are significantly different (*P* < 0.05). (a) Phylum; (b–d) genus.

Lastly, the influence on the abundance of intestinal flora was studied. As shown in Figure 6, sequencing the 16S rRNA gene in the samples, *Firmicutes*, *Proteobacteria*, and *Actinobacteria* were found to be dominant at the phylum level, while *Candidatus_Bacilloplasma*, *Candidatus_Hepatoplasma*, and *Acinetobacter* were the dominant species at the genus level. In addition, the results showed that the relative abundance of *Rhizobium* and *Rhodobacter* in the intestinal tract was significantly decreased (*P* < 0.05) in GP1000 and GP2000 compared with CN.

## 4. Discussion

### 4.1. Effects of Garlic Powder on Growth Performance

Many stimulant herbs are now widely used in aquaculture due to their various beneficial properties [12]. Garlic powder contains a variety of sulfur ether compounds and other volatile substances, and its natural fragrance, also known as garlic aroma, has a strong lure effect on animals, which can regulate the reflex activity of the olfactory and taste centers to promote intestinal peristalsis and the secretion of digestive enzymes and further improve the digestion and absorption of animal feed [13]. In addition to its unique and attractive pungent smell, garlic powder has the efficacy of promoting digestion and growth [14]. The addition of garlic increased the food consumption of shrimp (*Litopenaeus vannamei*) and improved the utilization of protein and amino acids; in other words, it improves the growth performance of shrimp [15]. Garlic is known to contain a variety of bioactive compounds, such as flavonoids, steroid saponins, phytosterols, and water-soluble nutrients, all of which plays an important role in the metabolic system and immune response [16]. Asian sea bass showed better growth performance after feeding garlic powder [17]. In this case, crabs in GP1000 and GP2000 presented a significant rise in WG and SGR. Therefore, we can speculate that garlic powder relies on its distinctive garlic aroma to achieve the effect of inducing food and promoting intake. At the same time, garlic powder may improve its growth performance by enhancing the nonspecific immunity and antioxidant capacity in Chinese mitten crabs.

### 4.2. Effects of Garlic Powder on Nonspecific Immunity

With the development of aquaculture animal nutrition research, more and more attention has been paid to the immune function of nutrients [18]. In aquaculture, garlic is often used to strengthen the immune system of aquatic animals [19]. And the reason why is that it can increase the number of lymphocytes and macrophages and stimulate lymphocytes to secrete more interferon and interleukin, etc., which can destroy pathogen cells and enhance the body's nonspecific immunity [20]. By interfering with certain working enzymes in pathogens, garlic powder can reduce the pathogenic effect of pathogens and prevent sensitive cells from absorbing toxins. At the same time, it has also been confirmed that garlic powder can improve nonspecific immunity as well as disease resistance in *Lates calcarifer* [17].

PO activity can be considered an indicator of the immune status of invertebrates. It serves as the one who resists invading pathogens, participates in phagocytosis, and promotes bacterial agglutination and clearance [21]. The *prophenoloxidase* (*proPO*) system is an important component of natural immunity in invertebrates, and some studies suggest that *proPO* may be an acute response to invasive bacteria in Chinese mitten crabs [22]. In this experiment, the activities of PO in GP1000 and GP2000 were increased compared with that in CN, and combined with increased expression of *proPO*, it was reasonable to conclude that garlic powder might improve the immunity of Chinese mitten crabs by activating the *proPO* system. LZM can break down the peptidoglycan layer of the bacterial cell wall, leading to the lysis of pathogenic bacteria, so it is considered as a major nonspecific immune indicator in invertebrates [23]. In order to enhance the activity of the immune defense mechanisms and improve the resistance to disease in aquatic commercial animals, immune stimulants and medicinal plant supplements are usually used to stimulate natural killer cells and LZM activity [24]. Previous studies showed that adding garlic to the diets of rainbow trout (*Oncorhynchus mykiss*), Rohu (*Labeo Rohita*), and Asian bass (*Lates calcarifer*) increased globulin content and LZM activities, hence improving its immunity [25, 26]. Adding garlic into the diet of crustaceans, like kuruma shrimp (*Marsupenaeus japonicus*), can increase the expression of the *lysozyme* and *Toll-like* genes [27]. Similarly, this experiment's results showed that the LZM activity along with the expression of *lysozyme* of GP1000 and GP2000 was significantly enhanced and the gene expression of *Toll-like receptor 1* was also significantly increased after the addition of garlic powder. The fact that garlic powder can increase LZM activity in serum may be associated with an increase in the amount of LZM secreted by phagocytes or the amount of LZM synthesized by each cell.

Phosphatase is a lysosomal enzyme that plays a decisive role in cell lysis and differentiation. As a typical hydrolase, phosphatase is mainly used to remove toxins and detoxify pollutants, which is beneficial to the immune system of crustaceans [28]. It is often used as a reliable indicator to evaluate the immune status of crustaceans, and ACP is involved in the killing and digestion of pathogenic microorganisms during the immune reaction. In this experiment, the content of GP2000 of ACP in serum was significantly increased compared with CN, which may be related to the fact that garlic powder can activate secretion levels of monocytes and macrophages, increase the content of immunoreactive enzymes, and stimulate the immune system in Chinese mitten crabs.

### 4.3. Effects of Garlic Powder on Antioxidant Capacity

Natural products, including garlic powder, are considered better antioxidants due to their minimal adverse effects [29]. Phenols and saponins are compounds that provide antioxidant activity in garlic which can inhibit the formation of free radicals, enhance the uptake mechanism of endogenous free radicals, and increase cellular antioxidant enzymes, such as GPx, SOD, and CAT [30]. Scientists used FRAP and DPPH methods to test that garlic has obvious antioxidant activity, and to prove that garlic can prevent oxidative stress and the biological molecule damage induced by glycosylation, follow-up experiments showed that the garlic extract of natural products have the characteristics of oxidation and glycosylation to protect the instability of the structure of the protein [31]. On the other hand, garlic powder is prone to Cope elimination reaction at room temperature, forming 2-propenesulfonic acid, which is a very effective antioxidant involved in maintaining redox equilibrium *in vivo* [32]. The antioxidants (flavonoids and sulfur) in garlic can significantly boost the stability of membranes and protect tissues from free radical-mediated toxic damage [33].

Intensives breed aquatics that will lead to environmental stress, resulting in highly toxic reactive oxygen species (ROS), so the antioxidant system is an important immune defense system of crustaceans [34]. ROS are highly unstable molecules that cause oxidative stress and tissue damage when they accumulate in cells [35]. T-AOC, GPx, SOD, and lipid peroxidation product MDA have been widely regarded as typical biological indicators to test stress in aquatic organisms [36]. T-AOC is a comprehensive index of antioxidant performance, representing its ability to metabolize oxygen-free radicals. GPx is a significant antioxidant enzyme that reduces peroxides to H_2_O and oxidized glutathione [37], which can reduce organic lipid peroxides, protect cells from oxidative damage, and plays an important role in eliminating excess free radicals and maintaining homeostasis. The present experimental results showed that the activity of GPx was significantly improved after the addition of garlic powder. We can reasonably surmise that garlic powder can react with glutathione rapidly to produce oxidized glutathione (GSSG), and activate the oxidative stress protection response of the body through the oxidative stress sensor system. T-SOD has similar biological functions to the above two enzymes, which can effectively remove superoxide anion free radicals and prevent such cell damage, so as to achieve the cellular oxidant/antioxidant balance. CAT's biological function is to promote the breakdown of hydrogen peroxide in cells so that it does not further produce the highly toxic hydroxyl radical. Hydrogen peroxide, which penetrates the majority of cell membranes, is more cytotoxic than SOD, which cannot. This explains why CAT plays an important role in maintaining the antioxidant system. According to the present experimental results, the activities of T-AOC, GPx, and T-SOD in serum and hepatopancreas were significantly increased, which illustrated the strong antioxidant properties that garlic powder possesses. MDA level is one of the final products of lipid peroxidation and an important indicator of oxidative stress [38], which can cause cross-linking polymerization of macromolecules such as proteins and nucleic acids, leading to cell death. The results of this experiment showed that compared with CN, the serum MDA levels of GP1000 and GP2000 in experimental groups were significantly reduced, indicating that adding garlic powder alleviated the oxidative stress damage of Chinese mitten crabs, which may be related to the antioxidant effect of garlic powder. This improvement in antioxidant performance may be caused by garlic powder inhibiting the production of free radicals, promoting endogenous antioxidant enzyme mechanisms, improving antioxidant enzyme activity, and reducing lipid peroxidation. The same results can be observed in European seabass [39].

### 4.4. Effects of Garlic Powder on Toll Pathway and IMD Pathway

Since invertebrates lack adaptive immunity based on immunoglobulin, resistance to pathogen invasion relies on innate immune responses [40]. There is no doubt that Toll and IMD pathways are two of the most important microbial sensing systems in invertebrates, which play an essential role in modulating the expression of antimicrobial peptides. Toll pathway is associated with innate immunity in Chinese mitten crab, and many Toll pathway-related genes, such as *TLR*, *MyD88*, *TuBe*, and *Dif*, have been confirmed in economic crustaceans [41]. *Relish* is considered to be a crucial gene in IMD pathway involved in regulating the host's innate antibacterial response [42]. The *ALF* has antibacterial activity, especially high antibacterial activity against gram-negative R-type bacteria [43]. In this study, the addition of garlic powder results in a significant increase in the expression of the antimicrobial peptides *ALF*, *crustins*, and *lysozyme*. Combined with the above results, garlic powder can regulate the Toll1/MyD88 pathway and IMD/relish pathway, activate antimicrobial peptide expression, and improve the immune capacity.

### 4.5. Effects of Garlic Powder on Intestinal Flora Structure

The intestinal flora system provides the first barrier against pathogens for intestinal health and ensures the continuation of normal physiological functions of the intestinal tract in animals. The results of this experiment showed that after garlic powder was added, unique OTUs appeared in the intestinal contents. After adding garlic powder, the decrease of ACE, Chao1, and observed species indexes indicates that the species richness decreases. The decreased Shannon and Simpson indexes reveal that the diversity of intestinal microbes in GP1000 and GP2000 had decreased. The separated PCoA showed that the composition of intestinal flora in GP1000 and GP2000 was different from that in CN. It was speculated that the addition of garlic powder changed the intestinal microbial composition of Chinese mitten crabs.

Scientific studies have shown that garlic has positive effects on fish health, including beneficial modifications to intestinal flora structure in crabs [7]. It is worth mentioning that in this study, the intestinal flora structure of Chinese mitten crabs is mainly phylum Firmicutes, followed by Proteobacteria, Actinobacteria, and Bacteroidetes. The same composition of intestinal flora was also mentioned in another study, and the main flora is also composed of Firmicutes, Proteobacteria, Actinobacteria, and Bacteroidetes [44]. In this study, the abundance of Firmicutes in intestinal contents of Chinese mitten crabs in GP1000 and GP2000 was increased, while Firmicutes can participate in the degradation of complex compounds, producing short-chain fatty acids, and stimulate the innate immune response of the host [45]. Therefore, it can be inferred that garlic powder can improve the immunity of Chinese mitten crabs by increasing the abundance of Firmicutes in the intestinal contents of Chinese mitten crabs. The antibacterial activity of garlic is derived from modification of lipid biosynthesis and RNA synthesis in bacteria that inhibit both gram-positive and gram-negative bacteria; for example, garlic is effective against gram-positive bacteria such as *Staphylococcus aureus* and gram-negative bacteria such as *Escherichia coli* and *Aeromonas salmonellae* [46–48]. In an experiment with the model organism *Caenorhabditis elegans*, it was found that DNA damage in intestinal cells of *Caenorhabditis elegans* may be caused by the production of ROS by *Rhizobium*, leading to defective nuclear division [49]. This study found that *Rhizobium* abundance significantly decreased in GP1000 and GP2000, suggesting that garlic powder may reduce the production of reactive oxygen by reducing *Rhizobium* abundance, thus improving antioxidant capacity. The genus *Rhodobacter* was mentioned in another study as the disease-discriminatory taxa [50]. That explains the significant decrease of *Rhodobacter* abundance in GP1000 and GP2000, which may be related to the disease resistance of garlic powder. The antimicrobial properties of garlic may prevent the colonization of pathogenic bacteria in the gut and influence the immune response by providing an ideal environment for the proliferation of beneficial bacteria in the body and by manipulating the intestinal flora of the host to favor beneficial microbial communities [51].

## 5. Conclusions

Summarizing scientifically, adding garlic powder could markedly enhance growth performance, nonspecific immunity, and antioxidant capacity, activate Toll pathway, IMD pathway, and proPO system, and increase antimicrobial peptide expression, while simultaneously improving the intestinal flora structure of Chinese mitten crabs. In this experiment, the recommended content of garlic powder in the diet is 1000 mg/kg. These positive results could provide the basis and novel insights for further research into whether additional performance improvements can be achieved by adding garlic powder to promote sustainable aquaculture.

## Figures and Tables

**Table 1:** Formulation and proximate composition of the basal diet (%).

Ingredients	Composition	Proximate composition	
Fish meal	23	Energy (Mj kg^−1^)	17.45
Shrimp powder	9.6	Moisture	10.30
Cottonseed meal	14	Crude protein	39.31
Rapeseed meal	14	Ash	16.53
Soybean meal	14		
Wheat flour	20		
Soybean oil	2		
Calcium biphosphate	2		
Sodium chloride	0.4		
Premix^1^	1		

^1^Supplied per kg diet the following minerals and vitamins: vitamin A, 10,000 IU; vitamin C, 100 mg; vitamin E, 100 mg; folic acid, 5 mg; vitamin D, 2,500 IU; vitamin K_3_, 2.2 mg; vitamin B_1_, 3.2 mg; vitamin B_2_, 15 mg; vitamin B_6_, 10.0 mg; vitamin B_12_, 0.016 mg; choline, 600 mg; biotin, 0.15 mg; inositol, 200 mg; Fe, 65 mg; Cu, 20 mg; Zn, 40 mg; Mg, 12 mg; Mn, 26 mg; Se, 0.16 mg; Co, 0.6 mg; I, 0.45 mg.

**Table 2:** Primer sequence for real-time PCR assays.

Genes^1^	GenBank id	Primer sequence, sense/antisense	Product size (bp)
*β-Actin*	KM244725.1	TGGGTATGGAATCCGTTGGC AGACAGAACGTTGTTGGCGA	101
*CAT*	GU361618.1	AATGAGGAAGAGCGGCAGAG TCGGGACAAACATGAAAGAAGT	110
*GPx*	FJ617305.1	ATGCCAGAGTTCACTAAGAGG AAGGGTCACAGCCAGGTC	165
*TLR1*	JX295852	ACTGTCTTGCTCGTCGTCTT CTTATTGGCATCTGCTTCCT	135
*TLR2*	KC011816	GCATACCAGGACGACGAACA TGAGCGAGGAGAGCTTGTTG	130
*Myd88*	KC019316	TGGCGTGCCGACTTGATT TGACCCACCAGACTTCCT	178
*TuBe*	KC011815	GGTGGGTTACTCCTGTGACG ACCCGCATCATCCATTCCAG	123
*Dif*	KC900086	CCCAATGTTTGTAGCACGGC CTGGAGAGGCTAGTGAGGGT	192
*Relish*	GQ871279.1	TGGGTGTTACGAGTGCACAA TGGTCAGAGTAGGGAGAGGC	118
*Crustins*	GQ200832.1	GCTCTATGGCGGAGGATGTCA CGGGCTTCAGACCCACTTTAC	116
*ALF*	HQ850572.1	ACGAGGAGCAAGGAAAGAAAG TTGTGCCATAGACCAGAGACTT	188
*Lysozyme*	JN416111.1	CTGGGATGATGTGGAGAAGTGC TTATTCGGTGTGTTATGAGGGGT	111
*proPO*	EF493829.1	CCATGTCATCATTGCAGCGG TGTACTTGTGCCAGCGGTAG	119

^1^ *CAT* = *catalase*; *GPx* = *glutathione peroxidase*; *TLR 1*, *2 = Toll-like receptors 1*, *2*; *Myd88 = myeloid differentiation factor 88*; *ALF* = *antilipopolysaccharide factor*, *proPO* = *prophenoloxidase.*

**Table 3:** Effects of dietary supplementation with garlic powder on growth and feed utilization in Chinese mitten crabs.

Items	Control	Garlic powder	
		1000 mg/kg	2000 mg/kg
Initial body weight (g)	20.66 ± 0.29	20.63 ± 0.32	20.84 ± 0.20
Final body weight (g)	54.45^b^ ± 0.73	58.83^a^ ± 0.60	59.21^a^ ± 0.65
Specific growth rate (%day^−1^)	1.73^b^ ± 0.02	1.87^a^ ± 0.03	1.86^a^ ± 0.02
Feed conversion ratio (g/g)	2.02 ± 0.11	1.77 ± 0.07	1.85 ± 0.04
Survival rate (%)	80.50 ± 0.03	87.50 ± 0.04	87.50 ± 0.02

Note: means not sharing the same superscript in each column are significantly different (*P* < 0.05).

## Data Availability

The datasets presented in this study can be found in online repositories. The name of the repository and accession number are as follows: National Center for Biotechnology Information (NCBI) Bioproject, https://www.ncbi.nlm.nih.gov/bioproject/, PRJNA865535. The other raw data supporting the conclusions of this article will be made available by the authors, without undue reservation.
